# Supplementary material for: DMRforPairs: identifying Differentially Methylated Regions between unique samples using array based methylation profiles
Source: BMC Bioinformatics. 2014 May 15;15:141. doi: 10.1186/1471-2105-15-141 (PMC4046028; doi:10.1186/1471-2105-15-141)
Supplement: Additional file 2 — DMRforPairs output for the comparison of A431-MCF7 and NA17018-NA17105. Please start from the HTML files in each folder. Available via the BMC Bioinformatics website. [file 1471-2105-15-141-S2.zip › 1394847754114233_MOESM2_ESM/A431_MCF7/figures/28633.pdf]

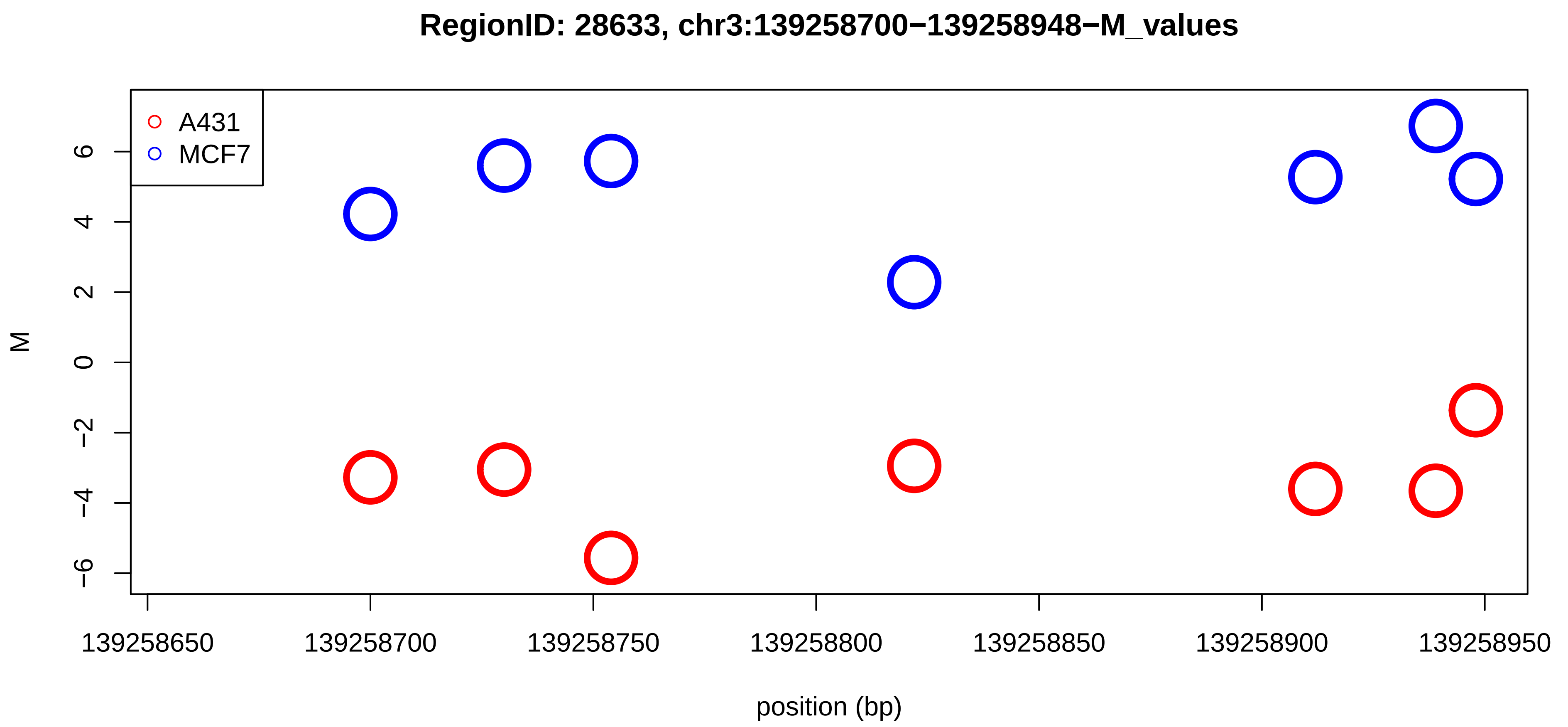

RegionID: 28633, chr3:139258700−139258948−M_values
A431
MCF7
M
−6
−4
−2
0
2
4
6
139258650
139258700
139258750
139258800
139258850
139258900
139258950
position (bp)

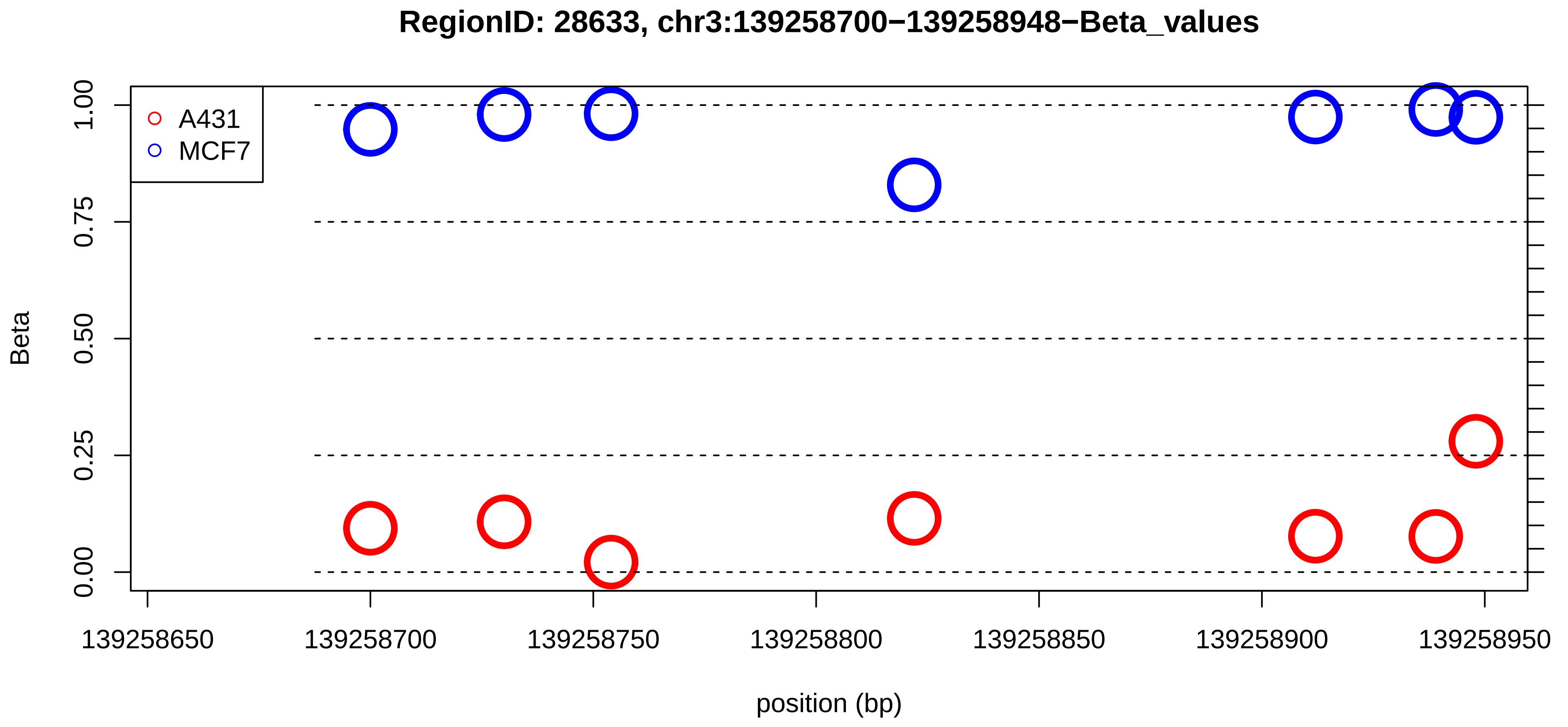

RegionID: 28633, chr3:139258700−139258948−Beta_values
A431
MCF7
Beta
0.00
0.25
0.50
0.75
1.00
139258650
139258700
139258750
139258800
139258850
139258900
139258950
position (bp)

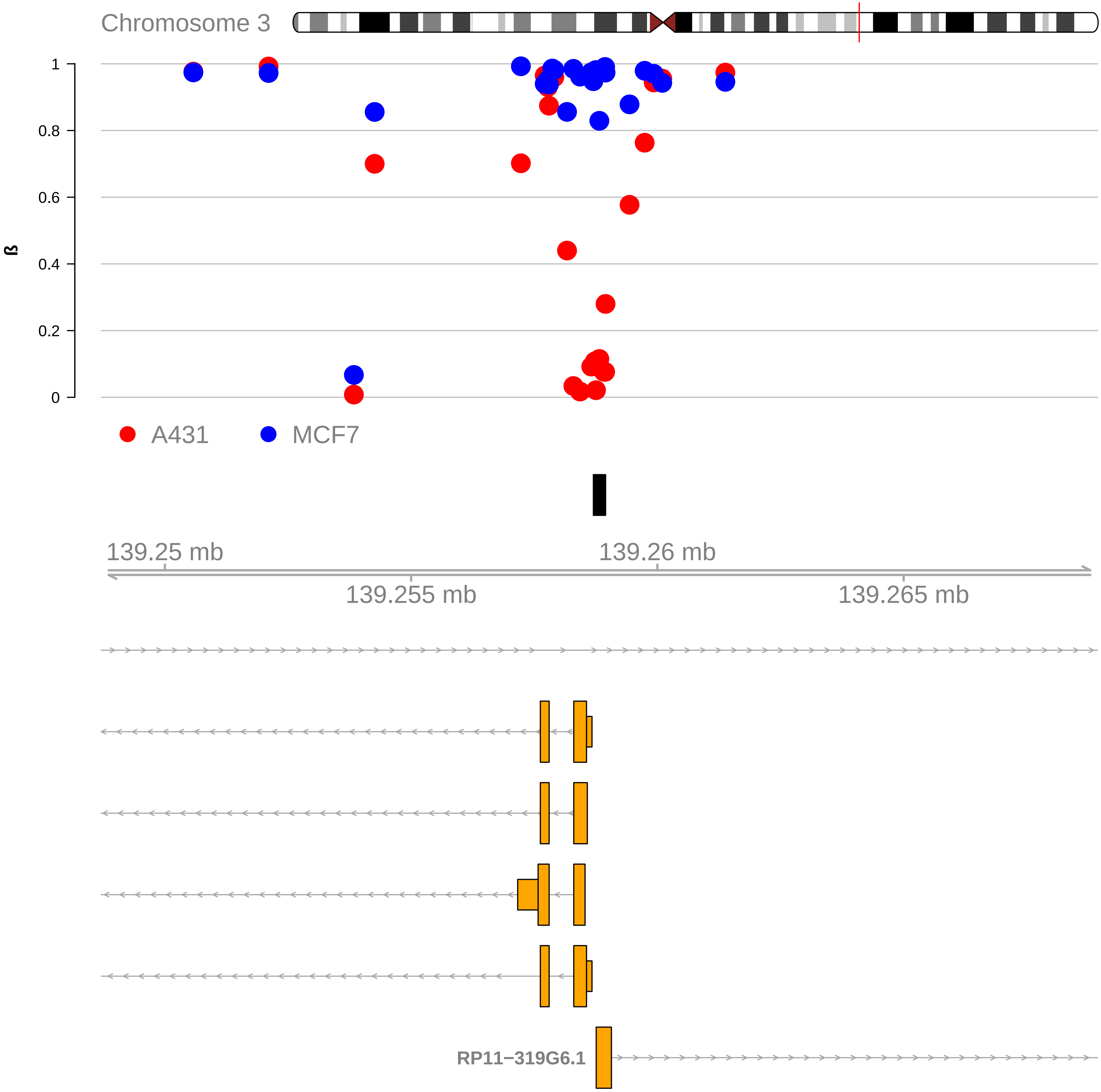

Chromosome 3
1
0.8
0.6
0.4
0.2
0
ß
A431
MCF7
139.25 mb
139.26 mb
139.255 mb
139.265 mb
RP11−319G6.1
